# A methodology for image-based measurement of plate movement in disengaged wet clutches

**DOI:** 10.1038/s41598-024-58012-y

**Published:** 2024-04-01

**Authors:** Lukas Pointner-Gabriel, Simon Flamm, Thomas Schneider, Karsten Stahl

**Affiliations:** School of Engineering and Design, Department of Mechanical Engineering, Gear Research Center (FZG), Technical University of Munich, 85748 Garching, Munich, Germany

**Keywords:** Edge detection, Object identification, Wet clutch, Plate movement, Drag losses, Mechanical engineering, Computational science

## Abstract

The drag loss behavior of a disengaged wet clutch is influenced, among other things, by the movement of the plates. Therefore, knowledge about the plate movement is essential for investigating and optimizing the drag loss behavior. This paper presents a methodology for image-based measurement of plate movement in disengaged wet clutches. A drag torque test rig is equipped with a camera to create the image series. The oil displacement from the measuring zone is crucial to obtain permanent optical access to the clutch pack. The rough plate positions are determined by segmentation using thresholding and template matching. Using the Canny edge detector significantly improves the accuracy of the position evaluation. The plate positions are finally converted into a metric unit based on the real plate thicknesses. The clearances are calculated from the determined positions of two adjacent plates. In the ideal case, an evaluation accuracy in the range of a few micrometers can be achieved. The image evaluation methodology is universally applicable to different clutch sizes, friction systems, plate types, and plate numbers. The methodology enables researchers to generate fundamental knowledge and derive design guidelines based on this, for example. In the development phase, it can also be used to optimize the design and operating parameters.

## Introduction

Wet-running multi-plate clutches (hereinafter referred to simply as wet clutches) are key components in modern drivetrain technology. However, in the disengaged state, drag losses occur mainly due to the shearing of the oil in the gaps. This subsequently leads to a considerable reduction in the overall efficiency of the drivetrain. Reducing the drag losses of wet clutches is the subject of numerous research projects.

In the disengaged state, the plates can move freely in the axial direction within the set total clearance. Consequently, the plates separate immediately after disengagement and then gradually re-position individually. The distance between two neighboring plates is called the clearance. Generally, the nominal clearance and the actual clearance have to be differentiated. The nominal clearance occurs in the case of an equidistant distribution of the set total clearance to the gaps. It is usually used as a theoretical parameter to describe the clutch configuration. The drag torque can be reduced by increasing the total clearance and, thus, by increasing the nominal clearance^1–3^. However, a wider total clearance also implies longer engagement times. The plate positions and, consequently, the actual clearances may vary during changing and constant operating conditions due to internal and external forces. The continuous changes in the plate positions usually result in an uneven distribution of the total clearance between the gaps^4^. The uneven and time-varying distribution results in an increased and varying drag torque^4^. Thus, the distribution of the total clearance between the gaps is a decisive factor influencing the drag loss behavior^5^. Therefore, measuring the plate movement is essential for investigating and optimizing the drag loss behavior. The drag loss behavior of a wet clutch depends on various other influencing parameters^1,3,6–8^. To the best of the author´s knowledge, the influence of plate separation and movement on drag loss behavior has not yet been fundamentally documented.

### Methods for measuring plate movement

Sensors and digital image processing algorithms have been used to date to measure plate movement. Beisel et al.^9^ used distance sensors to measure the axial movement of the outer plates. For this purpose, the outer plates were modified at several positions to include measuring points. The movement of the inner plates was not considered. Klausner et al.^10^ developed a Hall sensor to measure a point's axial and radial displacement on a plate. Additionally, strain gauges were applied on the plates to differentiate between rigid body movement and elastic deformation. Both test set-ups^9,10^ were used to investigate the occasional increase in drag torque at high rotational speeds. 3D plate movements can be captured simply by applying three sensors around the circumference. However, the measurement accuracy when using sensors within the wet environment is challenging to evaluate. In addition, the application of sensors requires modifications to the clutch components.

Albers et al.^11^ developed an approach to determine the plate positions based on image recordings. The approach proposes to detect the plates' vertical edges. During investigations on a prototypical drag torque test rig, interferences such as rotation-induced distortions and air bubble formation were identified. The approach is considered technically feasible, but its reliability is ultimately determined by the prevailing interferences caused by the oil. The used test set-up is not described in detail. Based on the approach of Albers et al.^11^, Markowsky^12^ implemented a plate position measurement on a drag torque test rig. A high-speed camera is used for image recording. This allows multiple images to be taken per revolution, even at high rotational speeds. In a preliminary study, various interference effects, such as the foaming of the oil or the formation of air bubbles, were identified. This makes image evaluation considerably more difficult. However, the exact image evaluation process and applied algorithms are not described.

### Objective

This study aimed to develop a methodology for measurement of the axial plate movement in disengaged wet clutches based on digital image processing. The methodology should be universally applicable to different clutch sizes, friction systems, plate types, and plate numbers. By applying the developed image evaluation methodology, future investigations can be performed, for example, on the influence of plate movement on drag loss behavior, the separation of the plates after disengagement, or the influence of different operating conditions on plate movement. The methodology enables researchers to generate fundamental knowledge and derive design guidelines based on this, for example. In the development phase, it can also be used to optimize the design and operating parameters. Due to its universal applicability, digital image processing is considered suitable for this purpose and is therefore used. The plate movement is recreated by sequencing the plate positions determined in the images of the image series. The detection of high dynamic effects was not followed. The setup is not intended for implementation in real applications since there is no technical benefit.

## Test set-up

Images with high resolution are required to accurately evaluate the plate positions. Since detecting highly dynamic movements is not an objective, there are no explicit requirements regarding the necessary frame rate. Therefore, an SLR camera (Canon EOS 700D) was chosen to record the image series. With this camera, a maximum frame rate of approximately five frames per second can be realized at the maximum resolution of 5184 × 3456 pixels (image format 3:2). By using a macro lens (Canon EF-S 60 mm f/2.8 Macro USM), images with reduced distortion can be taken. An LED spotlight with a maximum luminous flux of 36,000 lm is used to brighten the surfaces. The light is not pulsed and has a color temperature of approximately 6000 K. High light intensity without pulses is essential for consistent light conditions and short exposure times. A high-speed camera does not bring any relevant advantages for the possible investigations mentioned in Section "Objective".

The camera is installed on the LK-4 drag torque test rig (Gear Research Center (FZG), Technical University of Munich). A detailed description of the test rig can be found in Ref.^13^.

**Figure 1:**
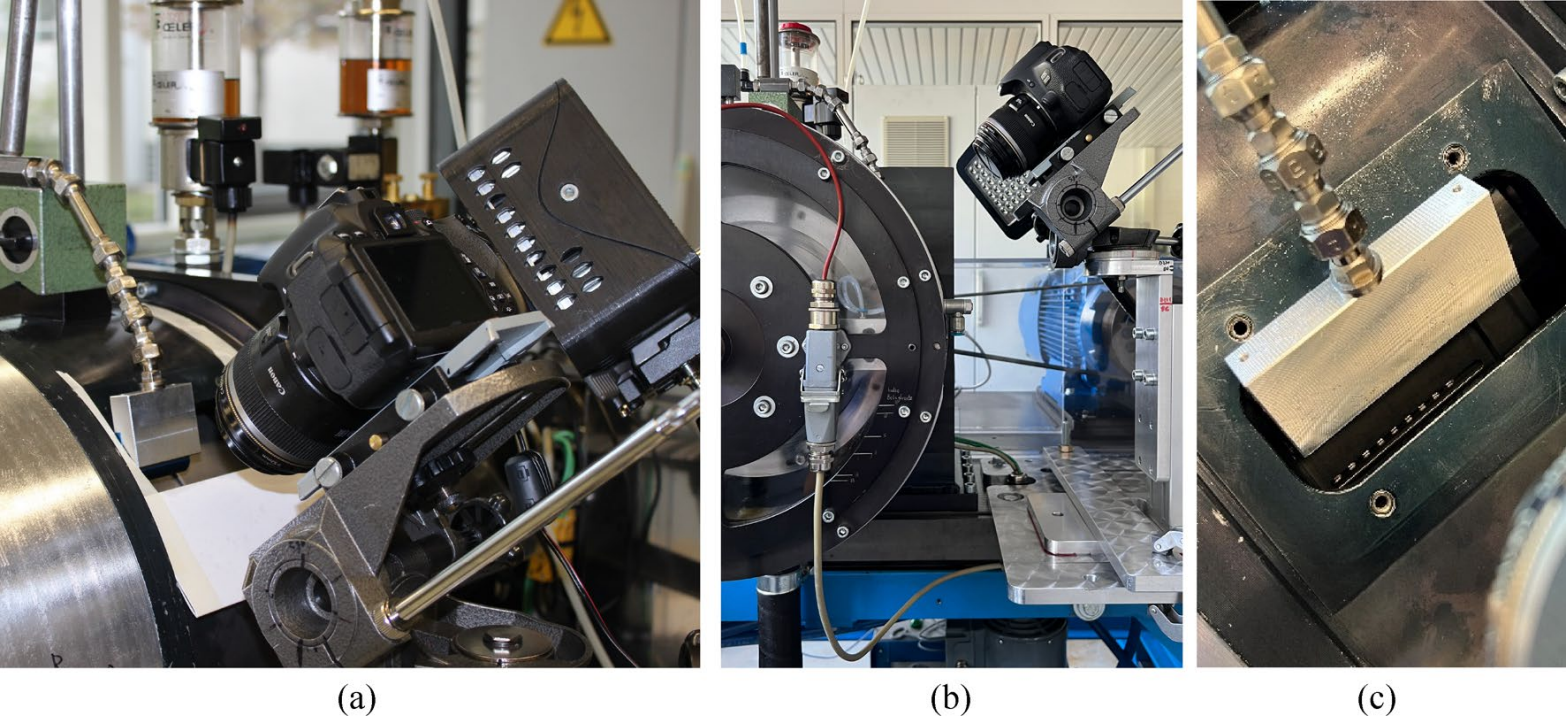
Test set-up for creating the image series: (**a**) Camera, LED spotlight, and flat-jet nozzle; (**b**) LK-4 drag torque test rig with the installed camera; (**c**) View of the clutch plates through the inspection window and a slot hole in the outer carrier.

**Figure 2:**
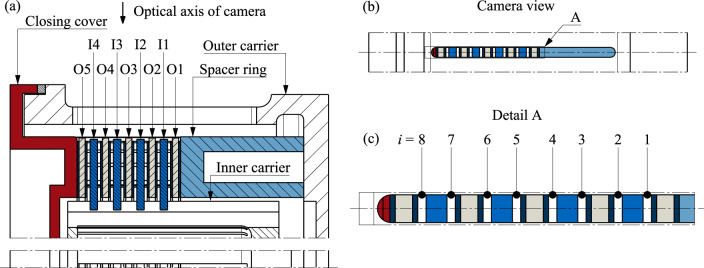
Mounting of a clutch pack of size D221: (**a**) Sectional view; (**b**) View of the clutch pack through the slot in the outer carrier in the direction of the camera's optical axis; (**c**) Detailed view of the relevant section with the numbering of the gaps *i*. Note: I, inner plate; O, outer plate.

The camera (see Fig. 1a) is attached to the test rig via an adjustable fixture (see Fig. 1b). The LED spotlight is placed to the camera's right (see Fig. 1a). However, due to the conveying effect of the clutch, the plates are covered with oil during operation (see Fig. 6). Therefore, compressed air is applied to displace the oil from the measuring zone. The flat-jet nozzle used (see Fig. 1a,c) is operated with a pressure of 2 bar. The pressure may need to be adjusted depending on the clutch system to be investigated. The width of the clutch pack gives the minimum width of the nozzle. The oil displacement by compressed air does not affect the plate movement and torque (see Section "Influence of oil displacement"). Optical access to the clutch plates is provided by an inspection window in the housing and the oil outlet openings in the outer carrier (see Figs. 1c, 2).

### Clutch systems and oil

Figure 2a shows an example of a mounted clutch pack consisting of five outer plates (O1 to O5) and four inner plates (I1 to I4) between the closing cover and the spacer ring. Figure 2b shows the clutch pack through the slot-shaped oil outlet in the outer carrier towards the camera's optical axis. Figure 2c shows the relevant section in detail. In this illustration, the total clearance is distributed equidistantly. The measurement of the plate movement is limited to brake operating mode with rotating inner plates, meaning the outer plates are stationary.

**Figure 3:**
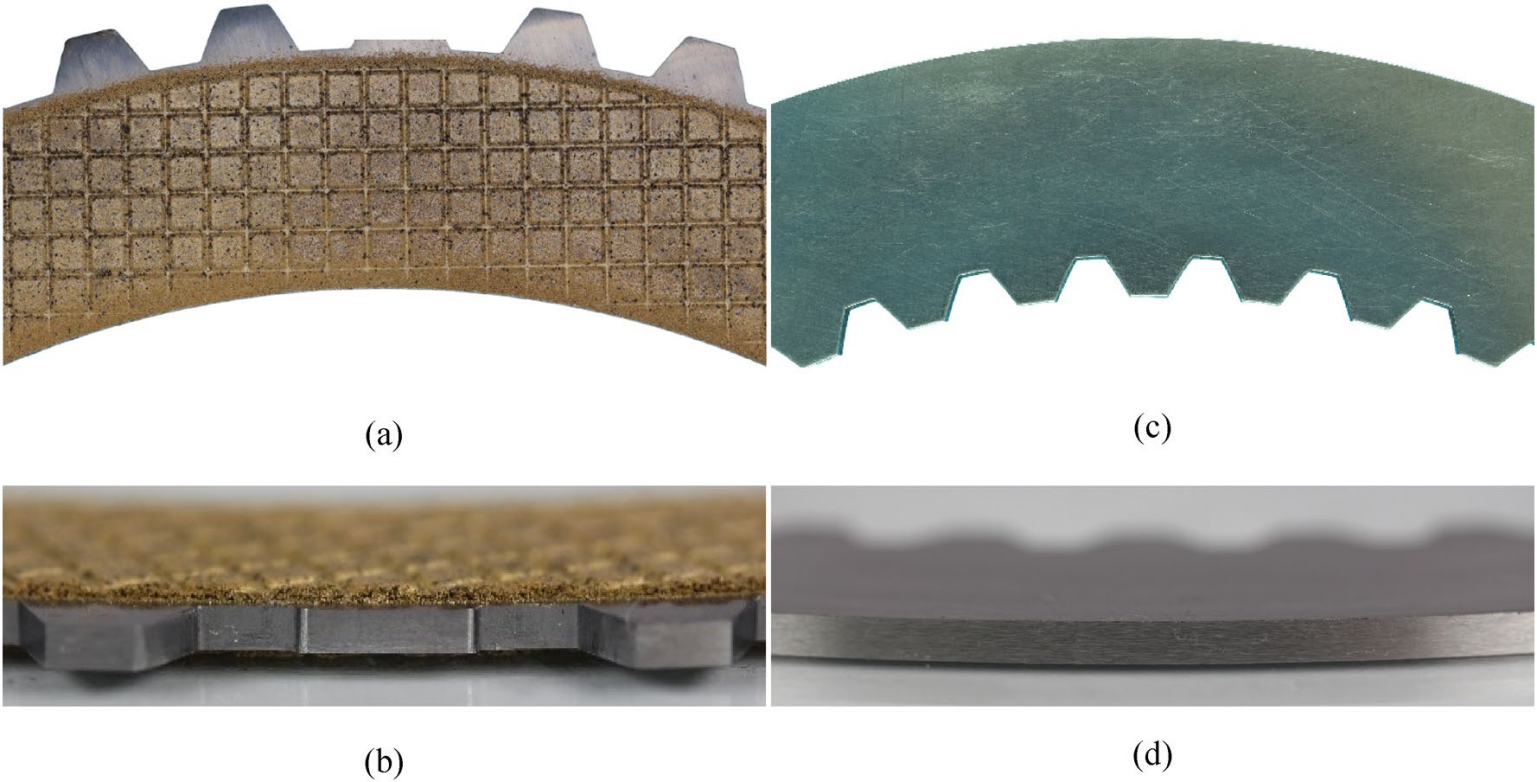
Close-up photographs of the friction plate (**a**) and (**b**) and steel plate (**c**) and (**d**) of size D221 in the top view and the radial view. Note: One tooth of the outer plate is removed to improve oil displacement (see Section "Modification of plates").

For the development of the image evaluation methodology, test image series were created with two clutch systems under different operating conditions. The D221 clutch size (see Fig. 3; mean diameter *d*_m_ = 221 mm) represents a clutch system from th industrial sector. The friction plate is designed as an outer plate, and the steel plate as an inner plate. The sinter friction lining of the friction plate has a waffle groove design. The steel plates can also be planar or waved. The D176 clutch size (see Supplementary Fig. 1; mean diameter *d*_m_ = 176 mm) corresponds to an automotive application. The friction plate is designed as an inner plate, and the steel plate as an outer plate. The paper-based friction lining is bonded to the core plate and has a waffle groove design. With the selected clutch systems, it was possible to generate various test cases with different clutch sizes, friction materials, plate variants, and plate numbers.

The physical characteristics of the oil used are listed in Table 1.

**Table 1:** Physical characteristics of the oil.

Density at 15 °C	840 kg/m^3^
Kinematic viscosity at 40 °C	26.8 mm^2^/s
Kinematic viscosity at 100 °C	5.6 mm^2^/s

## Image evaluation methodology

During image evaluation, the axial plate positions are determined from each image of the image series. The plate movement is then recreated using the plate positions and the image recording times. For determining the plate positions, all vertical edges are first detected, and then the plates in the image are identified. Subsequently, the edges are assigned to the identified plates. The plate positions represent the geometric center of the determined edge pairs with respect to a reference edge. Even though the plates can perform movements in the millimeter range, the clearances between the plates, on the other hand, are only a few tenths of a millimeter. For the D221 and D176 clutch sizes, a nominal clearance in the range of 0.1–0.3 mm is typical. A high evaluation accuracy is required since the clearances are calculated from the plate positions. Generally, it is recommended to choose the camera’s field of view so that clutch packs of typical dimensions fit into it. Considering this avoids changing the camera set-up when investigating different clutch dimensions.

### System characteristics and image features

**Figure 4:**
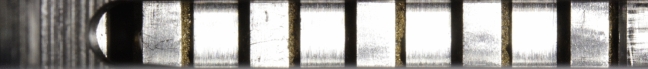
Image of a stationary clutch pack of size D221 in the end position.

The image evaluation methodology is described using images of a clutch pack of size D221 with five outer plates and four inner plates. As shown in Fig. 4, the images to be processed show, analogously to Fig. 2c, the outer and inner plates through a slot hole in the outer carrier between the closing cover (left) and the spacer ring (right). The friction linings of the outer plates are also visible. The friction lining on the left disappears in the shadow of the core plate due to the light coming in from the right. The clutch pack is in the end position in Fig. 4.

Numerous system and image features are used for image evaluation. The number of objects to be localized is the number of plates. The plates are arranged in a specified order and cannot overlap. The thickness of each plate can be measured in advance. The plate movement is small and limited by the set total clearance. The edges of the plates appear vertical when tilting is neglected. Brightness contrasts dominate.

Compared to the friction linings and the gaps, the inner plates and the core plate of the outer plates reflect more light. The strong intensity gradient at the transition from highly reflective surfaces to darker areas represents an additional image feature. Both features, in combination with *a priori* knowledge about the clutch system (geometry and behavior), would, in principle, be sufficient to determine the plate positions. However, due to different interference effects (see Section "Interference effects"), using only one of the mentioned image features is insufficient. Combining both features can determine the plate positions with acceptable accuracy and reliability, even in the presence of strong interferences.

### Interference effects

Various interference effects cause image evaluation to be challenging. The rotation of the inner plates causes distortions. Distortions also result from incorrect camera alignment^14^. In addition, distortions are caused by the lens^14^. The distortion resulting from the camera optics or alignment is considered during calibration (see Section "Main process") and compensated for if necessary. The projection plane of the camera must be aligned parallel to the subject plane to minimize the distortions.

**Figure 5:**
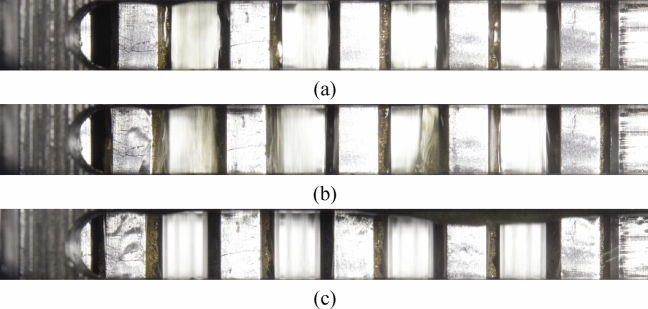
Different scenarios of oil coverage despite oil displacement: (**a**) At low rotational speeds, primarily rotating inner plates oil-covered; (**b**) In the range of aeration comparatively strong oil conveying; (**c**) At high rotational speeds, almost no oil coverage.

The plates may still be partially covered with oil despite applying compressed air. The oil coverage was found to depend mainly on the differential speed and the volume flow injected from the inside. Figure 5 shows three different scenarios of oil coverage. The inner plates are primarily covered with a continuous oil film in the low rotational speed range (see Fig. 5a). In the range of aeration, strong oil conveying makes image evaluation more difficult (see Fig. 5b). At higher rotational speeds, the plates are nearly free of oil (see Fig. 5c). It was found that nearly colorless oils considerably reduce interferences.

**Figure 6:**
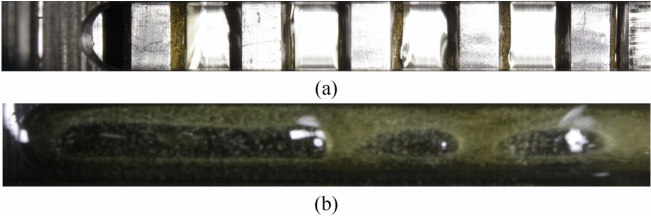
Different scenario of oil coverage without oil displacement by compressed air: (**a**) At low rotational speeds, inner plates partially oil-covered; (**b**) At high rotational speeds, plates completely oil-covered.

Figure 6 shows the importance of oil displacement. Due to the conveying effect of the clutch, the plates are completely covered with oil, especially at high rotational speeds.

### Modification of plates

The tooth of the outer plate, which is located in the measuring zone, hinders the displacement of the oil. Furthermore, the teeth cast shadows on the inner plates due to the light coming in from the right. The radial distance between the teeth and the inner plates also leads to perspective distortion and challenges in maintaining a required depth of field. The tooth in the measuring zone is removed to mitigate the interference effects. Removing the tooth improves the displacement of oil from the measuring zone. In addition, shadows and perspective distortions are avoided. Furthermore, removing any manufacturing marks (edges) on the lateral surfaces of the inner plates through cylindrical grinding, for example, is beneficial. This significantly reduces the detection of irrelevant edges in the image evaluation. At the same time, this increases the reflectivity of the surfaces and generates more distinctive brightness contrasts (segmentation, see Section "Main process").

**Figure 7:**
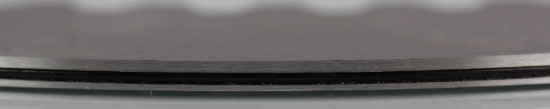
View of the lateral surface of a waved inner plate of size D221 with cylindrical groove and inserted O-ring.

In the case of a waved inner plate, the movement of the outer edges represents a superposition of the plate movement itself and the circumferential plate waviness. For this reason, the outer edges are not suitable for determining the plate positions. By adding a cylindrical groove in the lateral surface of the waved plate, a pair of edges is created that can be used in image evaluation. A black O-ring is inserted into the groove to improve contrast. Figure 7 shows the modification to a waved inner plate.

### Image evaluation process

**Figure 8:**
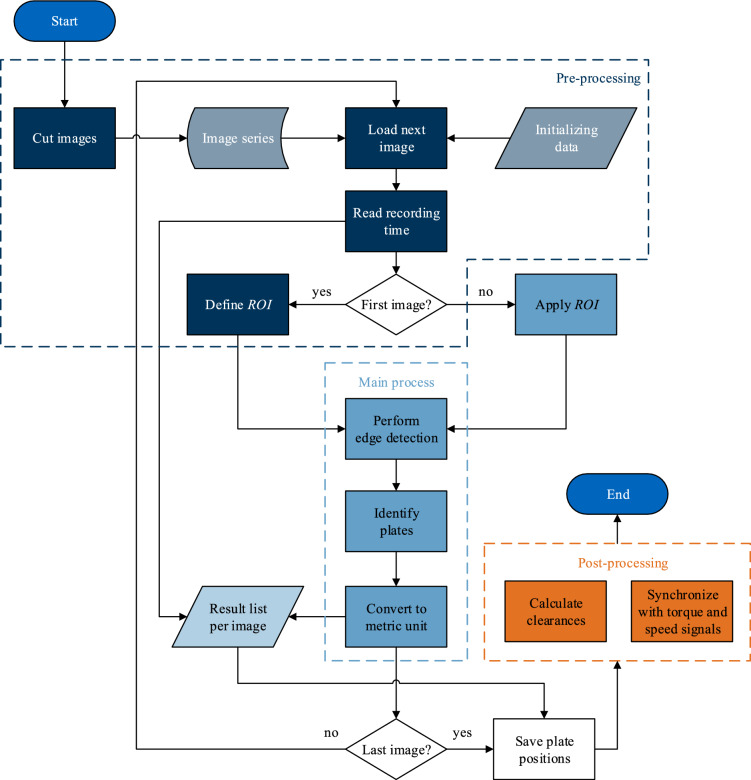
Image evaluation process.

The image evaluation process is shown in Fig. 8. The methodology is implemented in Python (version 3.9.7) and uses the open-source software and library OpenCV (version 4.5.1)^15^. The library matplotlib (version 3.5.1)^16^ is used to visualize the results.

#### Pre-processing

**Figure 9:**
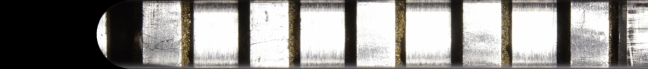
Image after applying ROI.

First, all images of the image series are vertically cut to the relevant region. This significantly reduces the file size and the evaluation time due to shorter data access times. Clutch-specific parameters such as the number and thicknesses of plates need to be specified for initialization. The recording time is read from the EXIF metadata of each image. The passed time between two images is determined by the difference in the time stamps. A Region of Interest (ROI) is defined manually in the first pass. Image regions outside the ROI are not considered in the image evaluation. Thus, irrelevant edge detections can be avoided. The defined ROI is applied statically to the entire image series. Figure 9 shows the defined ROI. The region highlighted in black on the left is ignored in the image evaluation.

#### Main process

The main process is performed chronologically for each image of the image series. In this way, evaluation results from the previous image can be used to evaluate the subsequent image. During the first pass of the main process, the detected edges must be manually assigned to the identified plates as part of the initialization in the initial image. Edges without assignments are rejected. The left outer edge of the stationary spacer ring is used as a reference for the positions (see the blue edge in Fig. 19). Also, templates of all plates are created in the first pass. The templates are used for plate identification using the template matching method (see Fig. 18).

##### Perform edge detection

**Figure 10:**
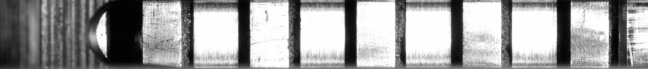
Image after conversion to grayscale.

The initial image in BGR color format must be converted to a grayscale image (OpenCV function *cv2.cvtColor()*). The grayscale image generated is shown in Fig. 10.

**Figure 11:**
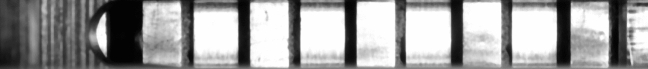
Image after applying a median filter.

Noise is removed using a median filter to improve the outcome of edge detection (OpenCV function *cv2.medianBlur()*). Compared to a linear filter, edges are better preserved using a median filter^14^. A 21 × 21 square kernel is applied. The image after applying the filter is shown in Fig. 11.

Then, as a final preparation for edge detection, Gaussian filtering (OpenCV function *cv2.GaussianBlur()*) is performed. Edge detection is performed using the Canny edge detector^17–19^ (OpenCV function *cv2.Canny()*). In contrast to other algorithms, the Canny edge detector does not provide a probability field but a binary mask of the detected edges. Moreover, the Canny edge detector returns unique positions of edges (width of one pixel)^17^. The result depends, among other things, on the thresholds of the Canny edge detector. Due to the high image resolution, the thresholds are set to low values (*th*_1_ = 15 and *th*_2_ = 50). Even with low variation of the thresholds, the edge detection result is robust.

**Figure 12:**
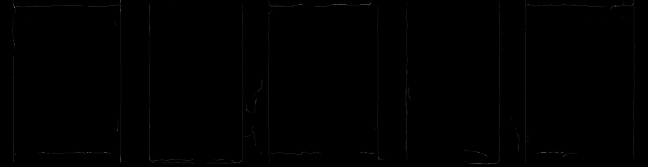
Detail of binary mask for two outer and three inner plates.

In addition, horizontal edges are using a Sobel operator^20–22^ (OpenCV function *cv2.Sobel()*). The horizontal edges are then removed from the previously determined set of edges. Figure 12 shows an example of the binary mask resulting from applying the Canny edge detector after removing horizontal edges for two outer and three inner plates. Nevertheless, some horizontal edges remain.

**Figure 13:**
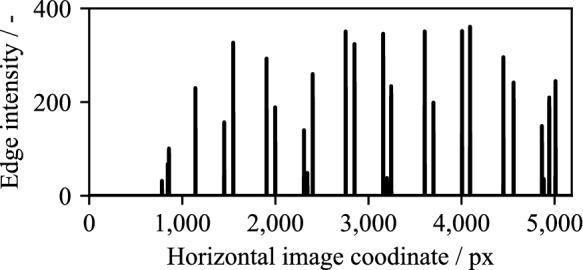
Distribution of edge intensities over the horizontal image coordinate.

Finally, the vertical edges are localized in the binary mask. For this purpose, the proportion of vertical edge pixels is evaluated via the horizontal image coordinate. This results in a distribution profile of the edge intensity over the horizontal image coordinate (see Fig. 13). The peaks in the distribution profile correspond to the vertical edges in the mask. The Hough transformation^23^ is not used because of the high computational effort required.

**Figure 14:**
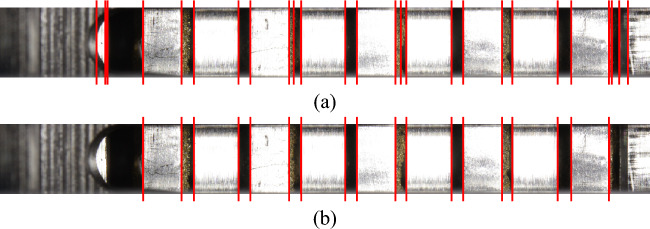
Set of detected edges before (**a**) and after (**b**) removing low-intensity edges.

The number of vertical edges to be detected is known from the number of plates. Any additional edges detected can be rejected based on comparatively low-intensity values. Finally, the edge positions are determined from the horizontal image coordinates of the maxima in the distribution profile. The edges detected in the image are shown in Fig. 14.

##### Identify outer plates: segmentation

**Figure 15:**
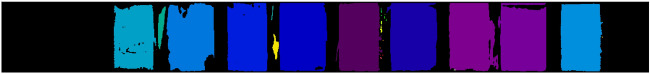
Segmented image after applying a global threshold.

In the next step, the plates are identified in the image. The strong light reflection of the plates makes them stand out clearly from the surroundings. This image feature can be used by thresholding methods that filter based on brightness values in grayscale images. Compared to the inner plates, the stationary outer plates are usually less covered by oil and can therefore be detected reliably. The expected brightness and the known thickness are used as criteria. To localize the outer plates, global thresholding (*th* = 130) (OpenCV function *cv2.threshold(cv2.THRESH_BINARY)*) is applied within the ROI (see Fig. 15). The segmentation divides the image into contiguous regions. Different colors indicate different segmented regions. For each outer plate, the largest contiguous region is determined within the plate position known from the previous image. It is checked whether the region's width and the outer plate's thickness match. For the first image of the image series, a manual initialization of the search region is performed during pre-processing. An outer plate is deemed localized if the match is within a defined tolerance. The correct outer plate is identified by order of instances in the image and comparison with the position of previous instance positions.

The detected positions are matched with the set of previously detected edges. The edges with the highest match now identify the detected outer plate instance. However, if no match can be found, the quality of the segmentation needs to be improved.

**Figure 16:**
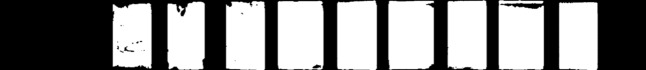
Binary mask after removing the quantile of 0.15 of vertically small regions.

The outer plates usually cover almost the entire height of the image (see Fig. 15). Small regions of the binary mask, such as between the first two plates on the left, are likely to represent erroneous detections. Therefore, regions below the empirical quantile of 0.05 in the vertical direction are not considered. If necessary, the empirical quantile is iteratively increased until a match between the width of the region and the thickness of the plate being searched for can be determined. Figure 16 shows the binary mask from Fig. 15 after applying a quantile of 0.15 (function *numpy.quantile()*).

Combining the identified positions and the detected edges completes the position determination of the outer plates.

##### Identify inner plates: segmentation and matching

**Figure 17:**
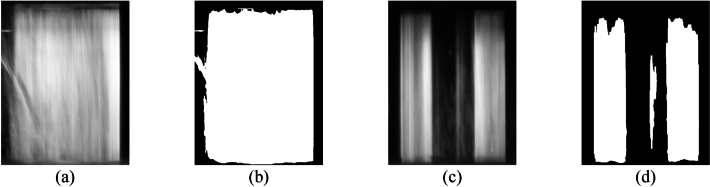
Comparison of greyscale input (**a**) and (**c**) and binary output (**b**) and (**d**). Visualization with planar (**a**) and waved (**c**) inner plate.

Since an inner plate is always located between two outer plates, the search area can be considerably limited, and each inner plate can be detected separately. The separate detection of the inner plates is advantageous since the oil coverage of the inner plates and, thus, the brightness of the lateral surfaces can vary considerably within one image. Therefore, a thresholding method that determines an optimal threshold is required. For this application, the method of Otsu^24,25^ (applied separately to individual regions) shows more robust results compared to adaptive thresholding methods with local thresholding (applied to the global image). Otsu's method is used to segment the plate being searched for from the darker surrounding area (OpenCV function *cv2.threshold(cv2.THRESH_BINARY* + *cv2.THRESH_OTSU)*). Figure 17 shows an example of two result masks after applying Otsu's method to images of a planar and a waved inner plate. As for the outer plates, the match between the width of the region and the thickness of the inner plate being searched for is checked.

In case of strong interferences, the width of the detected region may differ from the real thickness of the inner plate searched for. In this case, localization is performed using the more robust, but at the same, time more computing-intensive template matching method (OpenCV function *cv2.matchTemplate()*). During the first pass, templates of the lateral surfaces are created. Matching is performed on the binary mask.

**Figure 18:**
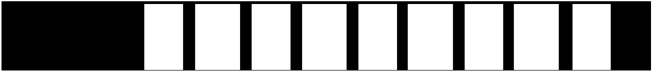
Templates generated from Fig. 4.

The positions of the inner plates determined via template matching are also matched with the set of detected edges; only if edges can be assigned to the detected position the plate identification is considered successful (Fig. 18).

##### Identify plates: final result

**Figure 19:**
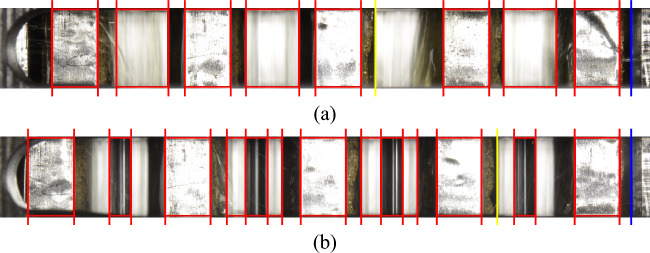
Graphical result representation of the plate detection: (**a**) Planar plates; (**b**) Waved inner plates.

Figure 19 shows the result of plate detection under comparatively strong interferences caused by the oil for planar and waved inner plates. Fully detected edge pairs are marked in red. Yellow marked edges indicate the detection of only one of the two plate edges. The reference edge (left edge of the spacer ring) is marked in blue. Accordingly, eight of the nine plates are localized, while one of the inner plates is only partially detected. The geometric center of an edge pair is finally specified as the plate position with respect to the reference edge.

##### Conversion and camera calibration

To convert the plate positions into a metric unit, a conversion factor *C*_*k*_ is calculated for each plate as the ratio of the real thickness *t*_*k*,mm_ in millimeters and detected thickness *t*_*k*,px_ in pixels. Table 2 lists real and detected thicknesses for plates of size D221 and the conversion factors. The global conversion factor is the arithmetic mean of all conversion factors. The first image of the image series at zero speed and without lubrication is used to calculate the global conversion factor.

**Table 2:** Measured and detected plate thicknesses, as well as conversion factors.

	Unit	Plate								
		O5	I4	O4	I3	O3	I2	O2	I1	O1
Thickness measured *t*_*k,*mm_	mm	2.50	2.98	2.48	2.98	2.48	2.98	2.49	2.98	2.51
Thickness detected *t*_*k*,px_	px	316	363	318	365	315	362	315	365	312
Conversion factor *C*_*k*_	µm/px	7.91	8.20	7.80	8.18	7.88	8.24	7.90	8.15	8.05

Note: Plate numbers analogous to Fig. 2a.

According to Table 2, the conversion factors *C*_*k*_ vary minimally for the outer or inner plates. The conversion factors *C*_*k*_ for the outer and inner plates form two groups of values. Such differences are due to the different subject planes of the outer and inner plates and, thus, different distances to the camera optics. No correction is made for these deviations since interferences caused by the oil dominate. For this reason, the minimal radial distortion caused by the lens is also not corrected.

**Figure 20:**
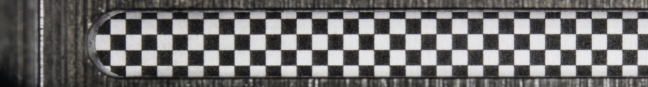
Calibration pattern.

Calibration and conversion could be performed using Zhang's method^26^. In this case, an exactly parallel and thus time-consuming alignment of the projection plane of the camera and the subject plane would no longer be necessary. Distortions can also be eliminated through correction factors. However, this method was not applied since the achievable accuracy is insufficient for the present task. Figure 20 shows the calibration pattern for applying Zhang's method^26^.

#### Post-processing

The plate positions are synchronized with the measured data in the final post-processing. The actual clearance *h*_*i*_ is calculated from the positions of two neighboring plates and their thickness. The clearances are only calculated if the edge pairs of two neighboring plates were detected in the image evaluation process.

## Application of image evaluation methodology

**Figure 21:**
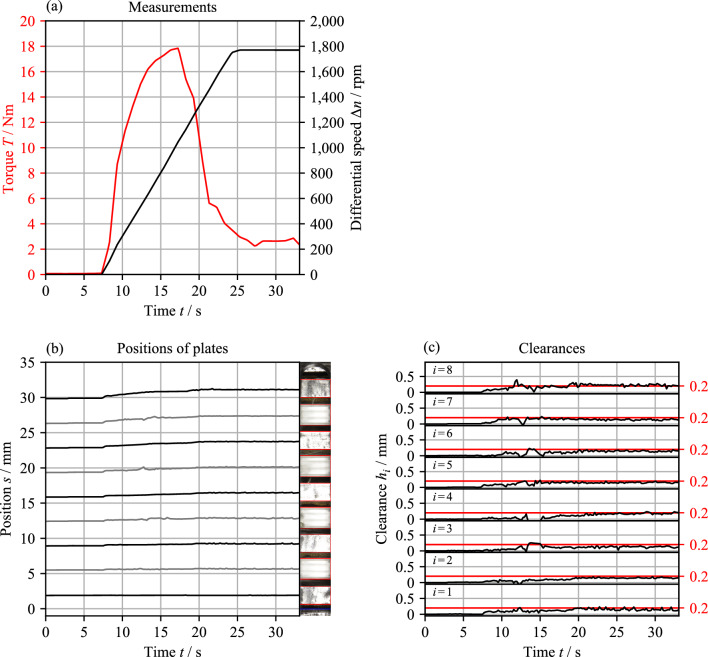
Exemplary measurement with a clutch pack of size D221 (planar plates, eight gaps, specific flow rate 0.5 mm^3^/s/mm^2^, nominal clearance 0.2 mm). Note: For the videos, see Supplementary Video 1.

Figure 21 shows an exemplary measurement with a clutch pack of size D221 with planar plates and eight gaps. The corresponding videos are shown in Supplementary Video 1. The total clearance was set to 1.6 mm. Thus, the nominal clearance is 0.2 mm. The plates were in the end position at the start of the test.

Figure 21a shows the applied differential speed profile and the resulting torque. Due to the test rig concept, the measured torque at non-constant speed additionally contains the acceleration torque from the inertia of the inner carrier and the inner plates. According to the CAD data of the clutch system, the acceleration torque at the angular acceleration of 100 rpm/s is approximately 0.5 Nm. The plates' movement and the series' last image are shown in Fig. 21b. Figure 21c shows the variation in the clearances. The automatic determination of the plate positions takes approximately 30 s for the entire image series consisting of 175 images. Pre-processing takes about one minute, regardless of the length of the image series.

The plates start to separate due to the constant acceleration of the inner carrier to a differential speed of Δ*n* = 1800 rpm. The total clearance is fully utilized and is evenly divided between the gaps. At a then constant speed, there are no significant changes in position.

To show the universal applicability of the methodology, exemplary measurements with a paper-based friction material (see Supplementary Fig. 2 and Supplementary Video 3), waved inner plates (see Supplementary Fig. 3 and Supplementary Video 4), and a high number of gaps (see Supplementary Fig. 4 and Supplementary Video 5) were performed.

### Influence of oil displacement

Tests were performed to evaluate the influence of the oil displacement on the plate movement and the torque. The clutch was operated at a constant differential speed of Δ*n* = 100 rpm. In this rpm range, image evaluation is possible without oil displacement (see Fig. 6). The compressed air was applied during the test. As the torque curve and the plate positions (see Supplementary Fig. 5) show, there are no changes due to the oil displacement. Thus, the oil displacement does not influence the measurements. The corresponding videos are shown in Supplementary Video 2.

## Limitations and outlook

The test set-up can only be used to create image series with permanent optical access to the clutch plates. For this reason, investigations are limited to brake operating mode with the outer carrier stationary. For investigations in clutch operating mode, at least angle-controlled triggering of the camera must be implemented. A ring-shaped spotlight situated coaxially to the camera’s view could be used to avoid shadows and, thus, improve image evaluation. Further, a telecentric lens could be used to capture images without perspective error. Currently, images are captured at only one physical position. This means that only the axial plate movement can be captured. By installing three cameras, the 3D plate movements could also be captured. In the range of very high speeds, elastic deformations of the plates can also occur^10^. However, it is impossible to distinguish between elastic deformation and pure rigid body movement from the images. The image evaluation methodology is, therefore, not suitable for investigations at very high speeds. Image evaluation is currently performed after the tests have been run. For real-time image evaluation, the data transfer capabilities need to be improved. Many of the mentioned additions require the usage of a dedicated image processing camera. In the case of strong disturbances caused by the oil, superimposed movements with amplitudes in the range of a few hundredths of a millimeter may not be detected. The main movements can be detected with sufficient accuracy regardless of the disturbances. The oil displacement needs to be improved to capture even superimposed dynamic movements. High integration of the camera set-up into the test rig could possibly enable alternative ways for oil displacement.

## Discussion

In contrast to the test set-up developed by Markowsky^12^, an SLR camera is used instead of a high-speed camera since the focus is not on detecting high dynamic effects. Moreover, using a standard SLR camera allows a low-cost test set-up to be realized. The main difference between the test set-ups lies in realizing optical access to the clutch pack. In contrast to the test set-up presented in this paper, the camera system is combined with a transparent displacer. The displacer can be positioned close to the measuring zone, gaining optical access to the clutch pack^12^. However, during the development of the test setup presented in this paper, it was found that, in particular, the oil-air mixture, which is present mainly in the high differential speed range, prevents continuous optical access. Thus, oil displacement by compressed air was chosen instead, guaranteeing optical access to the clutch pack up to high differential speeds. The provided supplementary videos prove the efficiency of the oil displacement. The developed image evaluation methodology for measuring the plate movement builds on the approach of Albers et al.^11^. The base concept for determining the plate positions is detecting the vertical plate edges, being described on a single image. Edge detection is performed based on contrast gradients. Previously, rotation-induced distortions are reduced by deblurring. No identification of the plates is implemented, which was found to be essential for the evaluation of the image series during the development of the presented methodology. However, essential improvements and extensions were required for automatic and reliable evaluation of image series of any length. The identification of the plates is crucial in order to assign the detected edges to the plates automatically. Using the Canny edge detector allows for determining unique positions of edges instead of a probability field, resulting in a higher accuracy. During the development of the presented methodology, blurring of the rotating inner plates was considered. However, it was found that this effect plays a minor role compared to other interference effects mentioned in Section "Interference effects". Hence, deblurring was not implemented in the image evaluation process.

Generally, the evaluation accuracy is defined by the image resolution and the length of the measuring zone in the axial direction. The maximum accuracy for the chosen measuring zone (see Fig. 4) and interference-free images is approximately 8 µm. To increase evaluation accuracy, the measuring zone can be reduced when investigating small-width clutch packs. However, in particular, in the differential speed range of aeration, strong oil conveying makes image evaluation more complicated (see Fig. 5), which may result in a slightly reduced evaluation accuracy. As stated in Section "Objective", the methodology aims to detect the main movements, not necessarily superimposed high dynamic movements of small amplitude. The main movements of the plates immediately after disengagement, e.g., are in the millimeter range (see Fig. 21b). Thus, the main movements can be reliably determined with sufficient accuracy regardless of the intensity of the interferences. In the case of investigating small plate movements, it is recommended to crosscheck the evaluation via the video to identify possible discrepancies.

A test run of one minute, for example, results in 300 images at a frame rate of 5 fps. In this case, the evaluation of the image series requires about one minute. Pre-processing takes about one minute, regardless of the length of the image series. In addition, oil displacement from the measuring zone is considered necessary to ensure permanent optical access to the clutch pack. The choice of camera type depends mainly on the objective of the investigations. Tracking algorithms were also considered for image evaluation.

## Conclusion

The developed image evaluation methodology combines standard image processing algorithms to measure the movement of the plates in a wet clutch. Canny edge detector, Sobel operator, global thresholding, Otsu's thresholding, and template matching are used to determine plate positions. The rough plate positions are determined via segmentation using thresholding methods and template matching. The accuracy of the positions is significantly improved by using the Canny edge detector. Based on the plate thicknesses, the position of each plate is finally converted to a metric unit. The actual clearances are calculated based on the detected plate positions. The image evaluation methodology is universally applicable to different clutch sizes, friction systems, plate types, and plate numbers. The oil displacement from the measuring zone is crucial for obtaining permanent optical access to the clutch pack. However, the remaining oil can still affect the image evaluation in specific rpm ranges. When using the presented set-up, the achievable accuracy depends on the selected measuring zone's size and the interference level. In the ideal case, an evaluation accuracy in the range of a few micrometers can be achieved. By applying the developed image evaluation methodology, future investigations can be performed, among other things, on the influence of different operating conditions on plate movement and drag loss behavior or the separation of the plates after disengagement. Thus, the methodology enables researchers to generate fundamental knowledge, derive design guidelines, and optimize clutch designs during development.

### Supplementary Information

Supplementary Information.

Supplementary Video 1.

Supplementary Video 2.

Supplementary Video 3.

Supplementary Video 4.

Supplementary Video 5.

## Data Availability

The image series used and analyzed during the current study are available from the corresponding author on reasonable request.
