# Supplementary material for: A methodology for image-based measurement of plate movement in disengaged wet clutches
Source: Sci Rep. 2024 Apr 1;14:7631. doi: 10.1038/s41598-024-58012-y (PMC10985100; doi:10.1038/s41598-024-58012-y)
Supplement: Supplementary file 1 — Supplementary Information. [file 41598_2024_58012_MOESM1_ESM.pdf]

A methodology for image-based measurement of plate movement in disengaged wet clutches

Lukas Pointner-Gabriel, Simon Flamm, Thomas Schneider, Karsten Stahl

Technical University of Munich, School of Engineering and Design, Department of Mechanical Engineering, Gear Research Center (FZG), 85748 Garching near Munich, Germany

Video recordings

Supplementary Video 1: Measurement with a clutch pack of size D221 (planar plates, eight gaps, specific flow rate 0.5 mm³/s/mm², nominal clearance 0.2 mm).

Supplementary Video 2: Evaluation of influence of oil displacement on drag torque and plate positions: Compressed air was applied as of t = 27.5 s (planar plates, eight gaps, specific flow rate 0.5 mm³/s/mm², nominal clearance 0.2 mm).

Supplementary Video 3: Measurement with a clutch pack of size D176 (eight gaps, specific flow rate 0.5 mm³/s/mm², nominal clearance 0.2 mm).

Supplementary Video 4: Measurement with a clutch pack of size D221 (waved inner plates, eight gaps, specific flow rate 0.5 mm³/s/mm², nominal clearance 0.2 mm).

Supplementary Video 5: Measurement with a clutch pack of size D221 (planar plates, 16 gaps, specific flow rate 0.5 mm³/s/mm², nominal clearance 0.2 mm).

Friction plate and steel plate of size D176

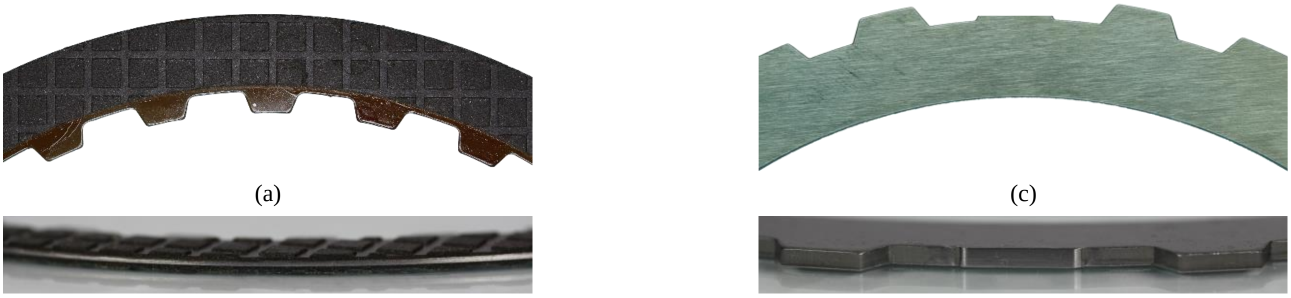

Supplementary Figure 1: Close-up photographs of the friction plate ((a) and (b)) and steel plate ((c) and (d)) of size D176 in the top view and the radial view. Note: One tooth of the outer plate is removed to improve oil displacement (see Section "Modification of plates").

Exemplary measurement – D176 clutch size

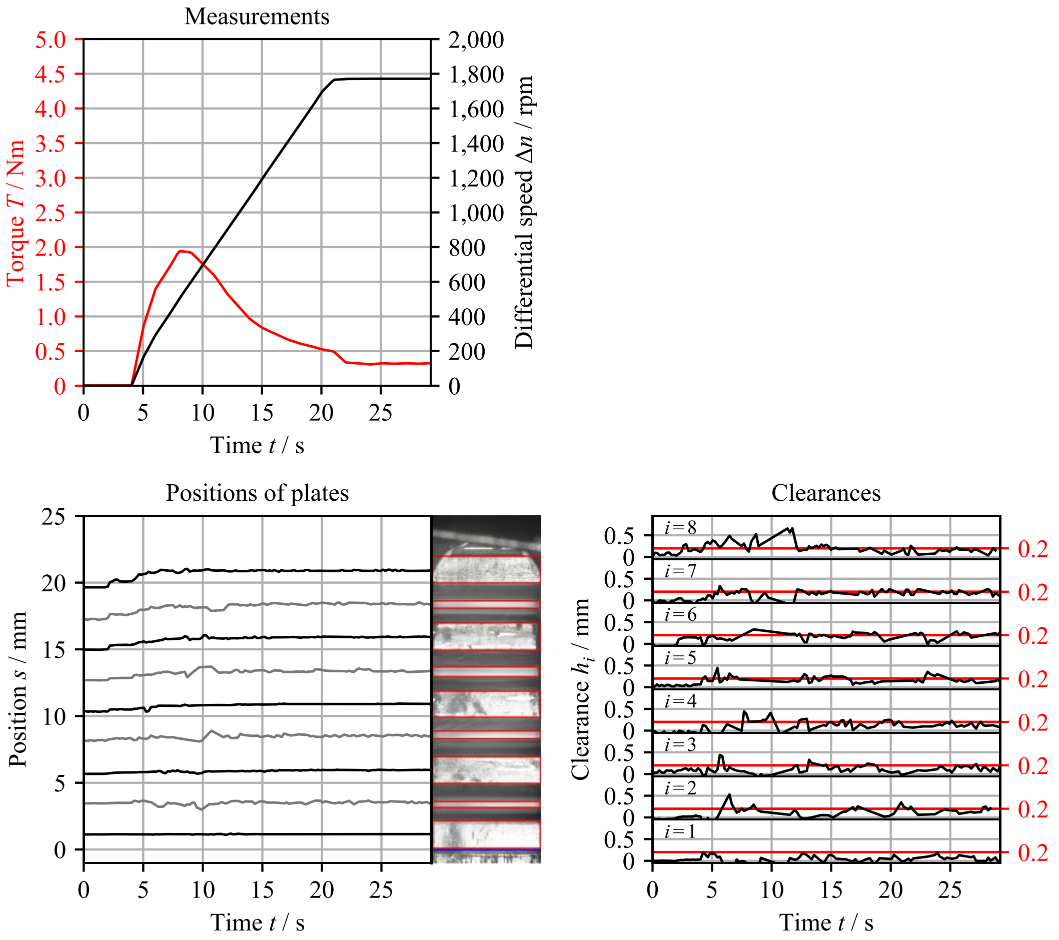

Supplementary Figure 2: Exemplary measurement with a clutch pack of size D176 (eight gaps, specific flow rate 0.5 mm³/s/mm², nominal clearance 0.2 mm). Note: For the videos, see Supplementary Video 3.

Exemplary measurement – Waved inner plates

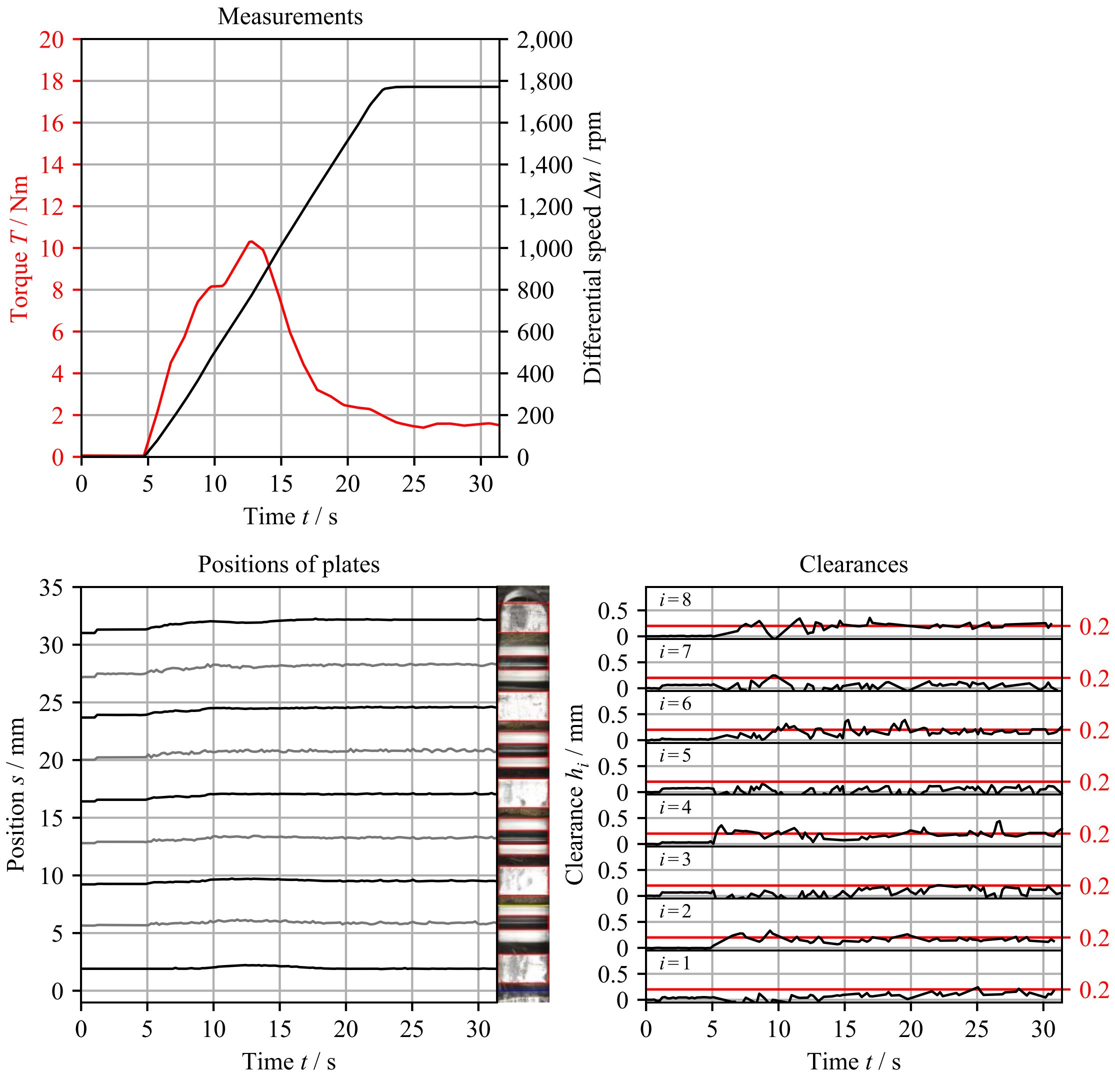

Supplementary Figure 3: Exemplary measurement with a clutch pack of size D221 (waved inner plates, eight gaps, specific flow rate 0.5 mm³/s/mm², nominal clearance 0.2 mm). Note: For the videos, see Supplementary Video 4.

Exemplary measurement – High plate number

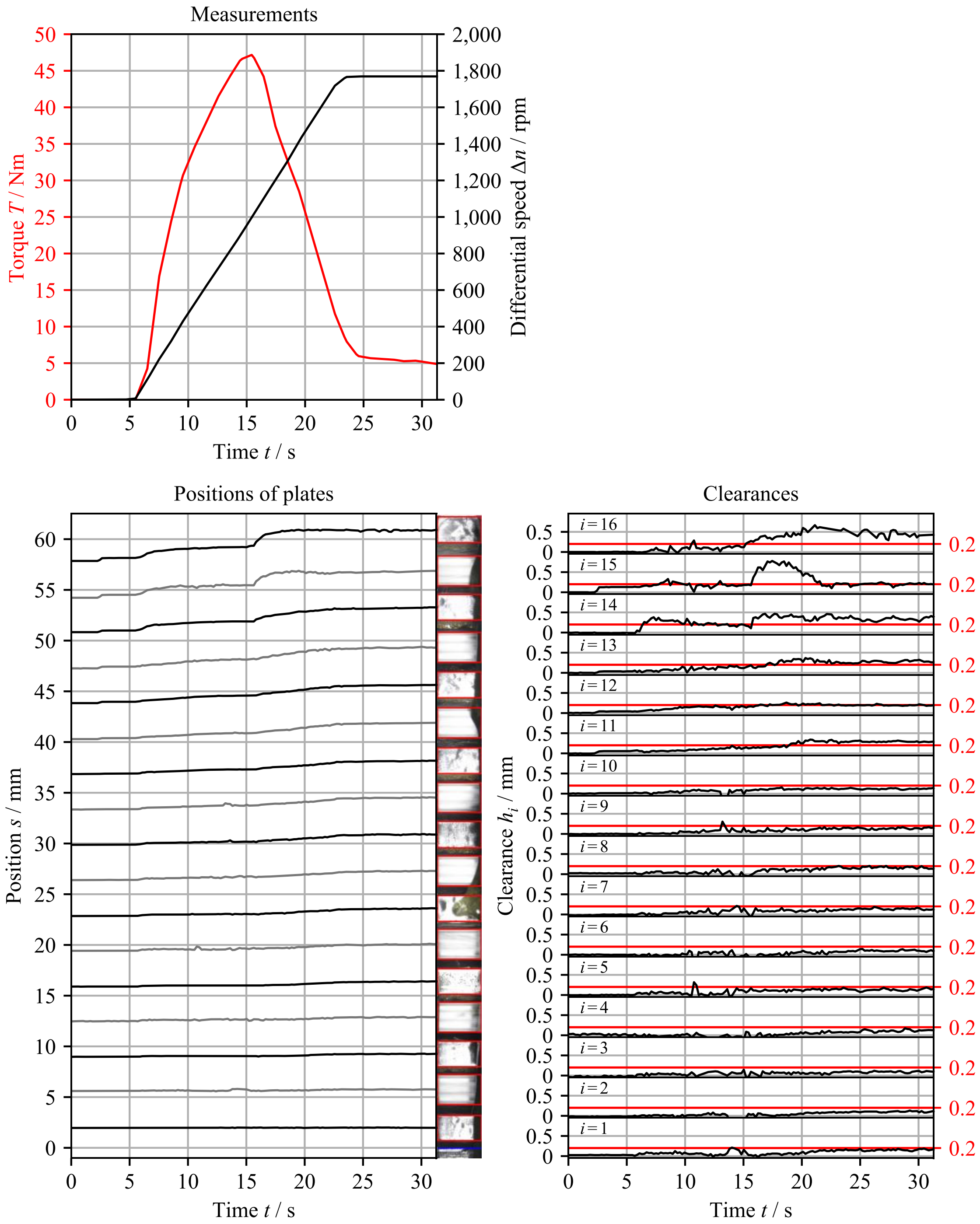

Supplementary Figure 4: Exemplary measurement with a clutch pack of size D221 (planar plates, 16 gaps, specific flow rate 0.5 mm³/s/mm², nominal clearance 0.2 mm). Note: For the videos, see Supplementary Video 5.

Influence of oil displacement

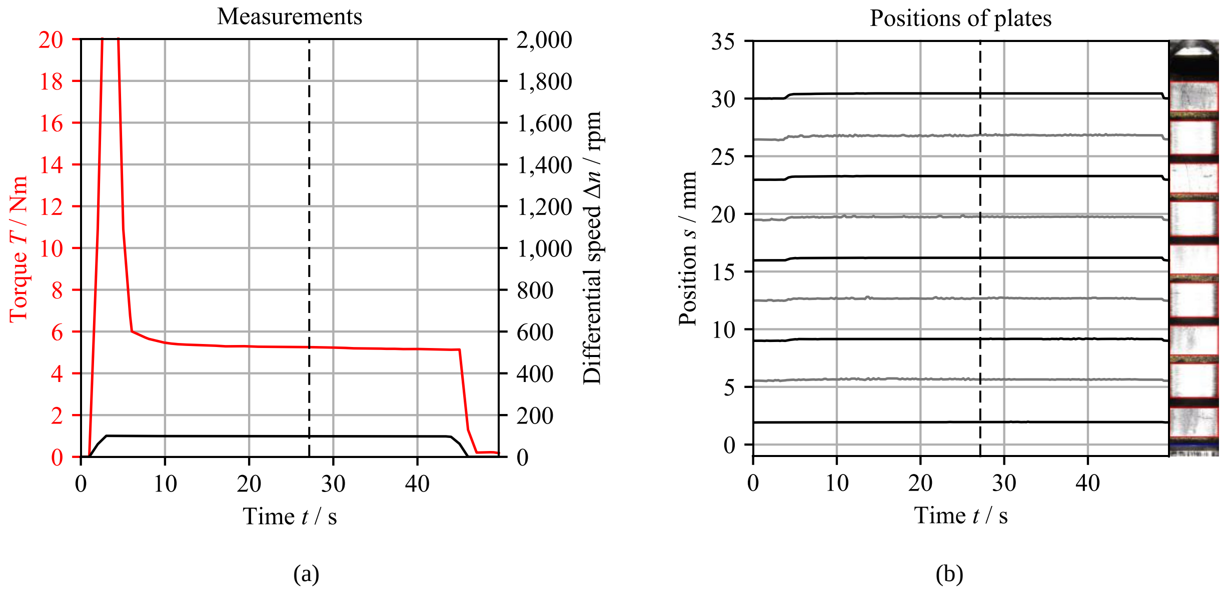

Supplementary Figure 5: Evaluation of influence of oil displacement on drag torque and plate positions. The compressed air was applied as of time $t = 27.5$ s. Note: For the videos, see Supplementary Video 2.
